# Phage Therapy in Veterinary Medicine

**DOI:** 10.3390/antibiotics10040421

**Published:** 2021-04-11

**Authors:** Rosa Loponte, Ugo Pagnini, Giuseppe Iovane, Giuseppe Pisanelli

**Affiliations:** Department of Veterinary Medicine and Animal Production, University of Naples Federico II, via Federico Delpino, 1, 80137 Naples, Italy; rosa.loponte@unina.it (R.L.); upagnini@unina.it (U.P.); giuseppe.iovane@unina.it (G.I.)

**Keywords:** alternative to antibiotics, bacteriophages, phage therapy, veterinary medicine, pets

## Abstract

To overcome the obstacle of antimicrobial resistance, researchers are investigating the use of phage therapy as an alternative and/or supplementation to antibiotics to treat and prevent infections both in humans and in animals. In the first part of this review, we describe the unique biological characteristics of bacteriophages and the crucial aspects influencing the success of phage therapy. However, despite their efficacy and safety, there is still no specific legislation that regulates their use. In the second part of this review, we describe the comprehensive research done in the past and recent years to address the use of phage therapy for the treatment and prevention of bacterial disease affecting domestic animals as an alternative to antibiotic treatments. While in farm animals, phage therapy efficacy perspectives have been widely studied in vitro and in vivo, especially for zoonoses and diseases linked to economic losses (such as mastitis), in pets, studies are still few and rather recent.

## 1. Introduction and History Notes

Bacteriophages are viruses that parasitize bacteria. This attribute can be used to treat infections caused by bacteria. Phage therapy is not a new practice. In fact, it has been used since the end of the nineteenth century, right after the discovery of bacteriophages and before the discovery of antibiotics [1]. Although Felix d’Hérelle is considered the father of bacteriophages and phage therapy, the first evidence of bacteriophages’ existence had been reported by English chemist Ernest Hankin, who described the bactericidal action of the waters of the Ganges and Jumna rivers on cholera, at the end of the nineteenth century. Hankin described the phenomenon without identifying the bacteriophages. About 20 years later, Frederick Twort noticed that plates were contaminated by microorganisms that killed bacteria. He theorized on the existence of bacteriophages but abandoned the theory because unsubstantiated [2]. On 10 September 1917, Félix d’Hérelle published a brief note in the prestigious “Comptes Rendus de l’Académie des Sciences”, in which he described a new kind of microbe as “an obligate intracellular parasite” of bacteria [3]. d’Hérelle showed that the viral particles were alive and capable of reproducing. Felix d’Hérelle’s discovery was the subject of many studies and contributed greatly to the knowledge of the therapeutic potential of phages. In 1923, Giorgi Eliava and Félix d’Hérelle founded “The Eliava Institute of Bacteriophages, Microbiology and Virology” in Tbilisi, Georgia. The Institute boasts developing several cocktails, but the most famous, produced by d’Hérelle, were the pyophage and the intestiphage, used for the treatment of purulent wounds and Enterobacteriaceae infections. In 1926, phage therapy was used by Pyle to treat salmonellosis in chickens (*Salmonella enterica* serotype *pullorum*), but the results proved to be a failure [4].

With the progressive production and massive use of antibiotics, the research into the therapeutic potential of bacteriophages was gradually neglected, especially from western Institutions. The work on this field continued only in Poland and the USSR, at the Eliava Institute in Georgia and at the Hirszfeld Institute in Wroclaw, Poland [5]. In the 1980s, about 60 years after Pyle’s results were published, the possibility of using phage therapy in animals was reconsidered by William Smith, who conducted experiments on chickens, cattle, and pigs [4].

## 2. Bacteriophages, Biological Characteristics and Classification

Bacteriophages are viruses that parasitize bacteria. They are essentially formed of nucleic acids (DNA or RNA) enclosed in a capsid of protein origin. As parasites, they need bacterial cells to survive and reproduce [1]. Bacteriophages are extraordinarily abundant in nature. It is estimated that there are 10^31^ phages present in the biosphere. Surprisingly, the total amount of bacteriophages is 10 times higher than bacteria [3]. They are also very ancient. It is estimated that they have been present on earth for more than three billion years [6,7]. Researchers estimate that bacteriophage-borne infections occur 10^23^ per second globally. The bacteriophages are abundant in human and animal organisms (in particular on their mucosal surface), in food [8] and in hostile environments, such as thermal springs of volcanic origin. Their virulence against bacteria is very specific [9].

Bacteriophages utilize two main types of replication: the lytic cycle and the lysogenic cycle. During the lytic cycle, the virus interrupts the physiological metabolism of the bacterial cell to facilitate the production of the bacteriophage progeny. After the viral replication, the infection results in the lysis of the host bacterial cell. Concerning the lysogenic cycle, after adsorption, the nucleic acid of the bacteriophage integrates with the genome of the host bacterial cell and produces a prophage where it is passed on to subsequent bacterial generations. Chemical or physical factors can activate the prophage, which can exist from the bacterial chromosome, thus starting the lytic cycle. A temperate bacteriophage has both lytic and lysogenic cycles [9]. Namely, the first cycle plays a key role in the therapeutic use of phage therapy. In fact, in phage therapy, “phage cocktails” are prepared by using lytic phages, which consist in the administration of viruses, which, during in vitro experimentation, show a lytic capacity against target bacterial pathogens [1].

According to the most recent classification [10] based on morphology and nucleic acids, all bacteriophages species are grouped in 11 orders (Belfryvirales, Caudovirales, Halopalevirales Haloruvirales, Kalamavirales, Levivirales, Ligamenvirales, Mindivirales, Petivirales, Tubulavirales, Vinavirales), each one divided into a different family, many subfamilies, and thousands of species. The Caudovirales order, which represents the only order to which all tailed bacteriophages belong, is the most representative one. In fact, this order includes 10 families, 44 subfamilies, 672 genera, and 1976 species. Of all the 10 families within the Caudovirales order, bacteriophages belong mainly to 4 families: Myoviridae, Siphoviridae, Podoviridae and Authographaviridae. From a morphological point of view, these families are distinguished based on the characteristics of the tail. Members included in the Myoviridae family possess a contractile tail. The viruses that fall into the category of Siphoviridae show a long noncontractile tail. Finally, the Podoviridae and Authographaviridae phages, previously included in the same family, are characterized by a short noncontractile tail and may be distinguished only from a genetic point of view because Authographaviridae phages show an RNA polymerase encoded by virion (RNAP) [10].

## 3. Crucial Aspects Influencing the Success of Phage Therapy

### 3.1. Collection of Samples and Isolation

To find bacteriophages, identifying the location of the bacterial hosts is paramount. To isolate phages from the bacteria responsible for skin infections, epidermal samples, secretions from healthy throats or exudates from wounds can be collected [11,12,13]. Similarly, for animals, to find phages against pathogenic bacteria for aquaculture fish, coastal waters or fish farm waters are collected [14,15]. Instead, phages against intestinal bacteria are isolated from fecal material, wastewater [16,17], or milk in the event of mastitis [18]. After specimen collection, pre-infection processes are critical before proceeding to phage isolation. The basic method of phage isolation was determined by d’Hérelle. The method he developed is described as an enrichment procedure [19]. Generally, the processing methods vary according to the type of sample. More specifically, in samples from seawater, phage count can be quite low. For this reason, processing the sample to obtain more concentration of phage population is necessary. To facilitate the concentration, filtering and precipitation actions are carried out, alone or in combination with each other [19,20].

### 3.2. Phage Resilience to Environmental Factors

Important characteristics to consider in phage therapy are the environmental conditions and phage resistance. External factors play a key role in the stability of bacteriophages. Neutral pH (6–8) is normally used to store bacteriophages for long periods of time, in solution or dried. Lower pH (4–6) usually reduces phage replication. In general, high temperatures do not affect phage survival. Most of them can survive in a range of temperatures between 40 and 90 °C. Some phages, such as the one infecting *Lactococcus*, can even resist the pasteurization temperature [21,22].

### 3.3. The Ratio between Phages and Target Bacteria

The Phages/bacteria ratio is an important factor to consider in phage therapy. Specifically, two main approaches are considered in phage therapy. The first one, the active approach, involves adding a small number of phages to the bacteria, and the elimination of the bacteria is related to the time needed to generate several generations of phages. In the second one, the passive approach, the phages are added to the bacteria in a certain amount to lyse bacteria in a short period of time. The passive approach seems to be more efficient than the passive one [22].

### 3.4. Accessible Diffusion to the Bacteria

Access to target bacteria is another fundamental aspect to consider in phage therapy. The structure and the composition of the surface where the bacteria are located can limit the success of phage therapy. In general, solid structure food like sliced turkey meat, lettuce leaves, or smoked salmon can reduce the diffusion of bacteriophages, probably because the target bacteria can be integrated into the food structure, which acts as a shield for phage spread [23]. In raw milk, phage K against *S. aureus* is inhibited. This may be due to the effect of some whey protein, which dampens the interaction with *S. aureus* and phage K [24]. In addition, there is evidence that bacteria can hide in particular sites of the host, protected from the phage. This problem is particularly evident in diseases caused by intracellular pathogens where the bacteria replicate in cells, which are inaccessible to the phages [22].

### 3.5. Monophage, Multiphage and Lytic Enzymes Preparations

Phage preparations can consist of only one type of phage (monophage) or multiple phages of different types (multiphage), also known as phage cocktails. In addition to being used for therapeutic purposes, monophage preparations can be used for the prophylaxis and biological control of germs. In this regard, an example of phage preparation as a food additive is Listex™ P100 (Micreos), an additive, which kept *Listeria monocytogenes* under control [25]. Generally, with the use of monophagic preparations, in an attempt to defend themselves from phage infection, host bacteria adopt strategies to neutralize the phage. As a result, phage therapy could be ineffective. Experimenting with a cocktail that recognizes only one bacterial strain could be costly and time-consuming but could represent a valid therapy against bacteria that are resistant to any other conventional therapeutic approach [26]. The use of multiphage preparations has proved to be very advantageous for several multiresistant bacterial species. A study investigated the use of a phage cocktail consisting of five different types of bacteriophages, which recognized a strain of *Klebsiella pneumoniae* found in infected wounds as a host and compared the effectiveness of this multiphage therapy with different monophagic therapies. Not only was the bacterial reduction much greater in cocktail-treated patients, but phage-resistant bacterial strains were also less likely to develop [27]. This shows how a cocktail consisting of phage and its mutants can increase the host range. The synergy action between phages in the cocktail is a very interesting feature due to which the activity of the first phage positively influences the activity of the other [26].

The lytic enzymes of bacteriophages allow these bacterial viruses to perform their lytic action. Although the characteristics of these molecules have been known for about 70 years, their possibility of use in therapeutic treatment started in the early 2000s. Holin and endolysin are the best-known lytic enzymatic proteins expressed by phages. They act on the lysis of the bacterial cell at the end of the phage infection. Unlike endolysins, holins are not able to establish cell lysis by themselves. For this reason, the only proteins taken into consideration in therapeutic practice are endolysins. In addition, these proteins are powerful, rapid in expressing their action, and function only on prokaryotic cells, so they have no effect on eukaryotic cells. A study has shown that lysine can reach and eliminate *S. Pyogenes* after passing the membranes of epithelial cells [28]. Lysine ABgp46 was recently discovered. It can destroy the cell membrane of several bacteria resistant to conventional drug treatments (such as *P. aeruginosa*, *A. baumannii*, and *Salmonella typhimurium*) [29]. Lysine administration was decisive in mice with bacteremia caused by strains of various bacterial genes known to be resistant to treatments with conventional antibiotic molecules [30].

The combination of phage therapy with other antimicrobial molecules could increase the bactericidal efficacy of a therapeutic treatment. In addition to showing antibiotic resistance, the bacteria can evade the phagic action, but if both therapeutic strategies were combined, the resistance mechanisms would occur more slowly, as the causative agent would need to fight with two different threats [31]. An effective combined antimicrobial treatment was the association of high doses of the antimicrobial molecule Kanamycin (lethal dose) with phage SBW25′2, which recognizes *Pseudomonas fluorescens* as a host [32]. The same phage combined with a lower dose of streptomycin (non-lethal dose) did not have the same effect. In contrast, resistance appeared. Combination therapy probably requires a lethal dosage of antibiotic molecules if combined with phages. Otherwise, attempts to avoid antibiotic resistance would prove to be completely fruitless [33]. The action of antibiotic molecules can be facilitated by the presence of depolymerases produced by phages that destroy bacterial biofilms. This combination produced encouraging results both for *E. coli* and *P. aeruginosa* [34,35]. A successful combination that helped reduce the appearance of resistance to antimicrobial molecules was the association of phage depolymerase enzymes with fluoroquinolone ciprofloxacin on a biofilm produced from resistant strains of *Klebsiella pneumoniae* [36].

### 3.6. Administration Route

Choosing how a drug is to be administered is a rather complex matter, which, in addition to requiring pharmacokinetics and pharmacodynamics, must meet the practical needs of the target population for which the preparation is intended. The same applies to phage preparations, for which various systemic and local routes of administration have been evaluated. One of the obstacles to phage therapy, for the usefulness of the oral route for phages administration, could be represented by gastric acidity. In fact, some bacteriophages can be inactivated by the acidic pH of the stomach. This obstacle can be overcome through buffer substances, which can neutralize the normally acidic environment of the stomach, such as sodium bicarbonate. In this way, the phage-based drug should be dispensed every eight hours before the main meals [37]. Various systems have been studied to allow the phages not to be inactivated by the enzymes present in the gastrointestinal tract. Micro capsulation is a technique, which has been used for the Felix O1 bacteriophage, included in an alginate–chitosan capsule (which cannot be digested). This technique has given the phage greater resistance to gastric pH [38]. Phage preparations can also be applied topically and are a rather useful route for the treatment of infected wounds in the form of gels or ointments. A preparation for local application was produced by the Eliava Institute in Georgia. The commercial name of the preparation is Phagebioderm. This product is useful for treating bacterial infections caused by *P. aeruginosa*, *S. aureus* and various species of the genus *Streptococcus*. Skin infections caused by *Salmonella* and *Campylobacter* in chickens can be effectively treated with phage preparations to be applied locally. Otitis is among the most frequent infection in both the human species (especially in children) and in animals. The possibility of therapy with bacteriophages also has been evaluated for this kind of infection, especially for those sustained by *Pseudomonas aeruginosa*. Single topical multiphage administration allowed for clinical and etiological healing [39]. The possibility of treating *Pseudomonas aeruginosa* bacterial otitis was also evaluated in dogs with encouraging results [40]. Other formulations, such as ocular and nasal suspensions, were studied. Another interesting formulation is the treatment of the airways by nebulization. In this way, the infections caused by bacteria that colonize the respiratory system and, in particular, the lungs can be treated [41]. Urinary infections are particularly frequent, and in these cases, the administration of the phage mixture (the pyophago, in this case) was evaluated directly in a patient’s bladder. The treatment was performed through a catheter for 10 days, twice a day [42].

### 3.7. Blood Diffusion and Antibodies Neutralization of the Phages

Important elements to take into consideration in phage therapy are also the blood diffusion of phages and the neutralization of phages by antibodies. Studies suggest that phages can be found in the blood of lab animals, after oral administration, in 2–4 h. In 10–12 h. can be found in internal organs. They can last in the body for several days, and there is no stimulation of the immune system, for example, cytokine production. The effect of bacterial reduction is only due to phage activity. Despite the ability of the phage to circulate in the blood, some authors showed that antibodies could neutralize them. However, this problem is not present during the administration of the first dose of phages because phages act very fast compared to antibody production. In a second administration, when the phage-neutralizing antibodies can interfere with the phages’ lytic action, the issue could be avoided by repeated phage administrations, by using an increased amount of phages or by the use of different phages [22,43].

### 3.8. Bacterial Resistance to Phages

The bacteria can also develop resistance to the phages through different mechanisms, such as loss of the phage receptors, which are proteins responsible for the attachment of the phages on the bacterial cells, degradation of the nucleic acids of the phage or a mutation of a gene that is important to phage replication. However, the speed of phage resistance developed by the bacteria is 10-fold lower than the speed of antibiotics resistance development. Furthermore, this resistance can be avoided by using more phages in one preparation or by isolating a new phage [43].

## 4. Phage Therapy in Poultry Farm

Antibiotic resistance is a phenomenon described with increasing frequency in poultry farms. Phage therapy could represent an alternative therapy to control the pathogens present in a poultry farm, also considering that there are restrictions on the use of antibiotic molecules to protect human and animal health. The potential use of phage therapy in chickens is not recent. Phage therapy was used to treat a systemic infection of *Salmonella enterica* (serotype *pullorum*) in chickens in 1926 when Pyle published the results of his research. Unfortunately, Pyle’s results were not very encouraging. Although in vitro, the phage preparation had demonstrated lytic activity against *Salmonella*, once administered to animals, there were no evident therapeutic effects, and there was no reduction in mortality compared to animals of the control group. At that time, it was not yet known that some bacteriophages could be inactivated by gastric acidity or enzymes present in the gastrointestinal tract. Therefore, the therapy had not had any positive effect [44]. The possibility of using phage therapy in farm animals (including chickens) was only considered 60 years later by Williams Smith [4].

### 4.1. Phage Therapy to Control Salmonella Infection in Poultry

According to the latest published EFSA report [45], salmonellosis has become the leading cause of foodborne zoonosis. A study published at the beginning of the 21st century by Sklar and Joeger on broilers showed that a phage preparation, capable of lysing a nalidixic acid-resistant *Salmonella enterica*, serovar Enteritidis strain (SeE Nalr), was able to reduce the bacterial load of *Salmonella enterica* ser. Enteritidis of a logarithmic base after two weeks of treatment [46]. To evaluate the efficacy of bacteriophages isolated from free-range chickens against *Salmonella enterica* ser. Enteritidis, one-day-old chickens were infected. After seven days, they were treated with the phage mixture orally. Researchers showed that orally administered phage mixture could reduce fecal contamination and consequently the spread of the pathogen and contamination of food of animal origin [47]. Researchers isolated more than 200 bacteriophages from various mediums between 2004 and 2005, and three of these phages, in particular, were able to inhibit *Salmonella enterica* ser. Enteritidis, Hadar and Typhimurium, in vitro. The three phages were then administered as oral monophagic preparations (with adequate antacid protection to protect the phages from acid digestion in the gastric environment) to chickens previously experimentally infected with *S. enterica* ser. Enteritidis. The first two phages reduced the colonization of the bacterium. The last phage, however, proved ineffective [48]. In another study, three bacteriophages isolated from wastewater from poultry farms were used to control infections caused by *Salmonella enterica*. Chickens were infected at 10 days of life with the germ, one day before the infection. The animals were treated with a mixture consisting of the three phages administered in drinking water or by aerosol. After 10 days from the infection, the animals were sacrificed to detect the presence of the germ or bacteriophages both from the gastrointestinal tract and in other organs. The phage treatment tested has proven effective in keeping *Salmonella enterica* infections under control, both administered as aerosol and orally [49]. The characteristics of the bacteriophage siphovirus PSE, isolated from poultry feces, have been evaluated both in vivo and in vitro to test its efficacy against *Salmonella enterica* ser. Enteritidis in quails. After administering the bacteriophage PSE in quails, through oral gavage and vent lip, an increase in the number of bacteria producing lactic acid and *Streptococcus* and a decrease in colibacilli was recorded in the experimental group compared to the control group. In another experiment, the possibility of using PSE as a preventive or therapeutic factor was evaluated. The results demonstrated that PSE administration as a preventive agent could reduce *S. Enteritidis* colonization more effectively than post-challenge administration. Furthermore, the resistance characteristics of the phage preparation to high and low pH, high temperatures, and bile salts, was also established, attesting to its ability to survive extreme conditions [50].

### 4.2. Phage Therapy to Control Colibacillosis Infection in Poultry

Colibacillosis, caused by avian pathogenic *Escherichia coli* (APEC) in poultry, can be responsible for high mortality. The respiratory tract infection is generally characterized by a fatal outcome. Once the germ has reached the air sacs, death from septicemia occurs [51]. After isolating bacteriophages against *E. coli* in wastewater and in poultry processing plants, a group of researchers evaluated the effectiveness of the phagic mixture obtained. In a first experiment, a group of three-day-old birds were infected with a concentration of *E. coli* (10^3^ CFU/mL) by injection into the thoracic air sac and simultaneously injected with bacteriophages (10^3^ or 10^6^ PFU). Another group was infected with a higher concentration of *E. coli* (10^4^ CFU/mL) and injected with a higher bacteriophage concentration (10^4^ or 10^8^ PFU). In another experiment, a group of one-week-old birds was instead subsequently treated orally with drinking water treated with 10^3^ or 10^4^ PFU of bacteriophage per mL. Subsequently, the birds were challenged with an air sac inoculation of 10^3^ CFU of *E. coli*. Alternatively, the water was treated with 10^4^ or 10^6^ PFU of bacteriophage per mL and birds were challenged with 10^4^ CFU of *E coli.* In a third experiment, one-week-old birds were infected by injection into air sacs of 10^4^ CFU of *E coli*. These animals were previously treated with water containing 10^5^ or 10^6^ PFU/mL of bacteriophage from one day of age to two weeks of age. The administration of bacteriophage through drinking water had no protective effect, as shown in the outcomes of the second and third research. On the contrary, simultaneous administration had reduced mortality by 25% in animals treated with the lowest concentration and by 5% in chickens with the highest phages concentration. In the animals belonging to the control group, the recorded mortality was 80%. These positive results, however, could be linked to the fact that the administration of the therapy took place simultaneously with the infection, so the bacteriophages had the opportunity to implement their cycle of lytic infection before the bacteria had the possibility to establish an infection in chickens [52]. Subsequently, the same research team experimented with the effectiveness of the use of bacteriophages (SPR02 and DAF6) to treat chicken colibacillosis through two different routes of administration: aerosol and intramuscular administration. In animals treated with a combination of the two phages DAF6 and SPR02 administered intramuscularly, the survival of chickens was greater than 80%, while in chickens belonging to the control group, survival was approximately 50% when the administration was performed within 48 h post-infection. The best route of administration, highlighted by this study, was the intramuscular route. Unfortunately, this is an impractical route of administration for the control of colibacillosis in intensive farms because it requires the individual administration of the mixture. Additionally, this administration route must be performed by specialized personnel, as it can cause muscle injuries resulting in damage to the carcass at the time of slaughter if performed incorrectly. The aerosol route, on the other hand, does not require specialized personnel and allows the simultaneous treatment of all animals but must be timely, i.e., within a few hours of infection. Otherwise, this route of administration is ineffective [53]. Huff and his research team subsequently published a study on combined bacteriophage and antibiotic therapy for the treatment of colibacillosis. After infecting 10 birds of 7-day-old with *E. coli* (6 × 10^4^ CFU) directly in the left thoracic air sac, the animals were treated immediately after the experimental infection with the administration of one of the two phages (DAF6 and SPR02) intramuscularly (3.7 × 10^9^ of phages DAF6 PFU for mL, and 9.3 × 10^9^ PFU for mL of phages SPR02). The antibiotic enrofloxacin molecule was administered at a concentration of 50 ppm in drinking water for 7 days after infection, starting immediately after infection. Mortality in the group of untreated animals had reached almost 70%, was 15% in animals treated with bacteriophages alone, 3% in animals treated with antibiotics only. The results were encouraging for the animals who received the combination therapy: no deaths were recorded. The authors suggested that combined bacteriophage and antibiotic therapy may increase the effectiveness of *E. coli* treatment in poultry [54]. Another research team explored the possibility of using a bacteriophage in the treatment of *E. coli* infections in chickens. In vivo bucket testing was conducted in groups of 12 broilers infected with *E. coli* H839E (1 × 10^8^ CFU) by injecting the left chest bag. The bacteriophage Phi F78E was administered at a concentration of 1.5 × 10^9^ PFU orally or by aerosol. The pathology score was lower in animals treated with bacteriophages compared to those not treated (2.5 vs. 4 in the treated and in the control group, respectively). Morbidity affected all animals in the control group, and only 60% of the bacteriophage treated. The mortality recorded was also lower in the group treated with bacteriophages (approximately 45%) compared to animals not treated with the preparation (approximately 75%) [55]. Other authors have evaluated the effectiveness of a spray phage preparation (SPR02) to treat the litter of chicken hens to prevent colibacillosis. The litter’s surface (3.9 m^2^) was contaminated with 200 mL from a culture of *E. coli* with a concentration of 2.8 × 10^8^ CFU/mL. The bedding of the phage-treated group was treated immediately after contamination with 200 mL of phage solution with a concentration of 8 × 10^8^ PFU/mL. The results of this study showed that the mortality of the control group subjects was 25%, while for the group treated with the phage suspension, it was 5%. The authors concluded that, if sprayed on the litter’s surface, the phage preparation SPR02 can help sanitize an environment contaminated with *E. coli*, reducing the mortality of the animals. Disinfection with phage-based products could be an effective strategy for controlling colibacillosis in intensive poultry farms [56].

*E. coli* infection is not only responsible for respiratory symptoms but also for lesions affecting the nervous system (meningitis) and septicemia. Some authors have evaluated the effectiveness of phage therapy also in this type of manifestations. In this case, the study was conducted in three-week-old chickens infected with an *E. coli* H247 K1 + strain by intracranial or intramuscular inoculation. They were then treated with coliphagus R at two different concentrations (10^4^ or 10^6^ PFU) through an intramuscular route. The survival of the animals treated with the coliphagus R was 100%, while none of the animals belonging to the control group survived the experimental infection. Preventive administration of the phage preparation 48 h before infection contributed to reducing mortality in 90% of the animals (1 bird out of 9 treated), while in the control group, the mortality was around 45% (4 animals out of 9 control). These results suggest that the proposed phage treatment could represent a valid alternative therapeutic strategy and may also be useful as preventive treatment [57].

### 4.3. Phage Therapy to Control Campylobacter Infection in Poultry

Infections sustained by the Campylobacter genus for several years have been the leading cause of foodborne zoonosis. In the latest EFSA report, they have lost the primacy but still remain among the most frequent causes [45]. This infection is particularly frequent in poultry, and poultry products represent the most frequent source of infection in humans. The infectious dose is rather low in humans, considering that 500 bacterial cells are needed to provoke the disease [58]. In 2005 the research results on the control of infections caused by *Campylobacter jejuni* in broilers were published, and the suitability of the preparation was evaluated from a therapeutic and preventive point of view. The mixture was administered orally. To test the effectiveness of the bacteriophage to prevent infections, the birds were treated preventively at 10 days of age, and after 4 days, they were experimentally infected. The therapeutic group was treated with the phage preparation for six days, starting five days after *C. jejuni* colonization of the broilers was established. In the therapeutic group, the data showed a reduction of 1 log in the count of *C. jejuni* compared to the control group [58]. Strains of *C. jejuni* and specific bacteriophages against the pathogen were selected from 205 broilers in the United Kingdom. The authors showed that the concentration of Campylobacter in the samples in which only the *C jejuni* without bacteriophages was present was higher in CFU/g by 6.9 log units, compared to the case where naturally occurring bacteriophages were present, was lower in CFU/g by 5.1 log units [59]. After isolating 53 phages from poultry stool samples, the same research team selected two bacteriophages, which had demonstrated good in vitro replication capabilities and had a wide host range (CP8 and CP34). They investigated their ability to control Campylobacter infections in poultry. The chickens were experimentally infected with *C. jejuni* HPC5, and GIIC8 isolates through the oral route from 18 to 20 days of age. After 5 days (at 25 days of age), the birds were treated with phage preparation, which was administered only once through the oral route. In treated animals, a reduction in the colony-forming units was recorded [60].

The bacteriophages that recognize *C. jejuni* as a host are divided into three groups according to the structural, genomic and specific receptor characteristics to which they bind to establish an infection against the host bacterial cell [61], probably the bacteriophages belonging to group II, to attach and establish the infection, can use different receptors present on the surface of the bacterial cell [62,63,64]. A research team assessed the efficacy of the CP220 bacteriophage, belonging to the second group of phages, to reduce *C. jejuni* in chickens experimentally infected with both *C. jejuni* and *E. coli*. The blind content of the birds was sampled after 48 h of infection and the single administration of phage CP220 of 7-log PFU, the colony-forming units of *Campylobacter jejuni* HPC5 had decreased by 2 log CFU/g [65].

The possibility of studying a combined treatment with bacteriophages of groups II and III was tested. 20-day-old chickens were infected with 10^9^ CFU of *C. jejuni*. After 7 days from the inoculation of the bacteria, the infected animals were treated with a suspension of 5 × 10^8^ PFU of CP14 (group III), CP81 (group III) or CP68 (group II), both in monophagic mixtures and in combination with a multiphagic mixture (administered by gavage or through food). On the fourth day after the therapeutic treatment (at 31 days of life), the animals were sacrificed by euthanasia. The count of the blind content of the animals treated with CP14 alone was 1 log10 CFU/g, superimposable on the control group. The combination of CP14 and CP81 did not favor any reduction in the bacterium count. A reduction of 3 log10 CFU/g was achieved when the CP68 replaced CP14 the next day. The significant reduction in *Campylobacter* colony-forming units is probably attributable to the possibility that phages must use different types of receptors. The consequences on the intestinal microbiota following the administration of bacteriophages in chickens have only recently been evaluated. After being infected with *C. jejuni* HPC5, the birds were treated with a mixture of two different bacteriophages. The blind content contained 2.4 log10 CFU g fewer bacteria than in the untreated group with the cocktail 48 h after administration [66].

In a recent study, Richards et al. used a mix of two virulent Campylobacter phages (CP20 and CP30) to treat broiler chickens previously colonized with *C. jejuni* HPC5. They found a significant reduction of Campylobacter counts from cecal contents, especially 2 days posttreatment, displaying a reduction of 2.4 log10 CFU g^−1^ relative to mock-treated Campylobacter colonized controls. They also found that the bacteriophage’s action had no negative effect on the intestinal microbiota and acted selectively only on *C. jejuni* [67].

Phage therapy has proven effective in reducing symptoms related to *Clostridium perfringens* infection. A study involving more than 900 chickens tested the possibility of using 5 phage cocktails at 10^5^ PFU/mL administered orally (through drinking water or by oral gavage) in animals between 0 and 42 days of age infected with *Clostridium perfringens.* The authors showed that the reduction in mortality following phage treatment was 92% compared to untreated animals. One of the cocktails administered (INT-401) also contributed to increasing the growth indexes, such as the increase in loss (WG) and the food conversion index (FCR), compared to the animals in the control group [68].

## 5. Phage Therapy in Bovine Species

Studies on the use of phage therapy in the bovine species were conducted mainly to control mastitis. Mastitis is a vastly common pathological condition that poses serious economic problems for cattle farms. The economic losses experienced by farms are not only ascribable to the cost of the pharmacological treatments required but are mainly related to the fact that the milk produced by cows with mastitis must be discarded. Often, the course of hyperacute mastitis could lead to the death of the animal or, as is the case with repeated mastitis infections, will end in lower production in quantity and quality. For this reason, farmers often decide to slaughter the affected animals or anticipate the dryness of the animals, which led in both cases to a loss on the total production of milk that can be sold. The most common pathogens implicated in the onset of mastitis are staphylococci, streptococci, and enterobacteria. Mastitis-causing pathogens can originate in the environment or be transmitted between infected animals. The microorganisms that are considered contagious, which have the mammalian gland of the infected cow as their main reservoir, are *Staphylococcus aureus* and *Streptococcus agalactiae*. The environmental pathogens, on the other hand, are those present in the surrounding environment of the cow, such as *Streptococcus uberis* and *E. coli* [69].

As early as 1980, a bacteriophage, phage K, was used to treat bovine mastitis caused by *S. aureus*, which unfortunately was not effective in controlling the infection. However, despite the poor efficacy, the bacteriophage was reevaluated about 25 years later. A study was published in 2005 on the ability of phage K to inhibit, in an in vitro assay, the emerging drug-resistant *Staphylococcus aureus*, isolated from samples of hospitalized patients and other *Staphylococcus* species isolated from bovine infections. The study showed that in an in vitro assay, the phage K can inhibit several species of *Staphylococcus* like *S. aureus*, *S. epidermidis*, *S. saprophyticus*, *S. chromogenes*, *S. capitis*, *S. hominis*, *S. haemolyticus*, *S. caprae*, and *S. hyicus*. Furthermore, its lithic action was also active on *S. aureus* methicillin-resistant strains (MRSA) isolated in the past years from hospital patients. Noteworthy, the authors demonstrated that the less sensitive MRSA strains to the lithic action of the phage K, after several passages, of the phage K, on the MRSA target strains will acquire the ability to lyse them efficiently [70]. The same research group isolated two other bacteriophages (CS1 and DW2) from sewage. The CS1 and DW2 bacteriophages resulted capable of lysing *S. aureus*. After producing a phage cocktail consisting of the two new isolated and phage K, the preparation was administered intramammarily. In terms of therapeutic efficacy, the results were encouraging, meaning that the phage preparation reduced the *S. aureus* and could be implemented into teat-washes as prophylaxis against mastitis caused by *S. aureus* [71]. An in vivo study aimed at investigating the possibility of using phage therapy in Holstein cows with staphylococcal mastitis by intramammary inoculation of a preparation based on phage K showed that the experimental therapy was not effective in the reduction of *S. aureus*, compared to the control group, probably due to the inhibitory effects of raw milk. Interestingly they found, in healthy quarters of animals treated with phage preparation, an increase of somatic cell counts (SSC), while in the quarters of animals, which were infected with *S. aureus*, any increase of SSC was observed. This opened a question on whether a specific or innate immune response against phage K could trigger somatic cell recruitment. Clearly, more studies are needed to better understand mammary gland immune’s response to phage treatment [72]. In a study published about 10 years ago, 8 temperate phages were isolated against *S. aureus*. Although they did not have lytic potential, two of them (ΦH5 and ΦA72) inserted in the same ratio (1:1) in the preparation were able to inhibit the proliferation of the *S. aureus*. Combining two phages was more effective than using a single one, probably because it prevented the selection of bacteriophage-insensitive mutants. However, even in this case, the bacteriophages had not been able to efficiently inhibit the proliferation of germs in the partially skimmed milk, while it had been more efficient in the milk subjected to heat treatments (both subjected to pasteurization and UHT) [73]. In 2012, the bacteriophage MSA6 was isolated from a cow with mastitis, similar in morphological terms to phage K, which was capable of killing several bacterial strains of *S. aureus* (both human and bovine strains) [74]. A year later, 10 bacteriophages were isolated from mastitis cows against *S. aureus*, these bacteriophages were able to inhibit several *S. aureus* strains, but above all, they were thermostable [75]. A group of researchers subsequently isolated the SPW lytic phage from samples of wastewater from a cattle farm [76]. This bacteriophage had excellent characteristics; besides the fact that it could kill several strains of *S. aureus*, it was stable to the variation of pH and temperature and resistant to isopropanol and chloroform.

Bacteriophage SA is a lytic bacteriophage recently isolated from wastewater. This bacteriophage also showed certain stability if subjected to different pH s and temperatures. It expressed its best lithic action if the pH was 7 and the temperature was 37 °C [77]. The lytic potential of bacteriophage SA and two other bacteriophages (SA2 and SANF) were investigated against 10 strains of *S. aureus* and one strain of *Micrococcus*. The bacteriophage SA displayed that it could lyse a wider host aspect than the other bacteriophages, while A2 was more efficient in inhibiting the growth of *S. aureus* in pasteurized milk. Bacteriophages USA 012 and USA 039 are lytic bacteriophages against *S. aureus*. They were isolated from cows with clinical signs of mastitis, which had demonstrated the in vitro ability to kill *S. aureus* strains and also methicillin-resistant strains). In vivo experiments have not been conducted on cows presenting mastitis but on mastitis model in mice. It was observed that the bacteriophage USA 012 had inhibited the proliferation of the bacterium and reduced the clinical signs of inflammation affecting the mammary gland. Two other bacteriophages were recently discovered: bacteriophages SAJK-IND and MSP. The former was isolated from wastewater, while the latter from bovine mastitis specimens [18]. The isolated bacteriophages were stable at a wide pH range (between 4 and 9) and were inactivated if subjected to a temperature higher than 60 °C, while the first bacteriophage was able to perform its lithic action on 120 different strains of *S. aureus* (i.e., 100% of the tested strains), the second bacteriophage was able to lyse just under half of the *S. aureus* strains under examination (48%).

Proteins of phage origin, such as endolysins, proved efficient in the treatment of mastitis. A recent study characterized endolysin encoded by λSA2, and B30 bacteriophages investigated their potential for the control of streptococcal mastitis. In an initial in vitro test, the authors observed that phage endolysins demonstrated a good lithic ability when subjected to pH conditions, ion concentrations that were similar to those present in cow’s milk. The best results were obtained when the two molecules were combined together. The lysine of the λSA2 bacteriophage showed a better lytic activity compared to the B30 in the presence of *Streptococcus uberis*, *Streptococcus dysgalactiae*, and *Streptococcus agalactiae*. In fact, the reduction observed was for all three bacterial species between 2 and 4 logs. In a mouse model experiment, both molecules of phage origin contributed to the inhibition of bacterial cell growth, even if the bacteriophage B30 showed a weaker lytic action against *S. dysgalactiae*. While synergy between the two molecules combined together was observed in vitro, in the mouse model, the synergy was not observed [78].

The possibility of using a phage lysine derived from bacteriophage K, CHAPk, was recently investigated for its antimicrobial properties and for the prevention and destruction of the biofilm during breast infections caused by *S. agalactiae*. Phage endolysin expressed not only a lytic action but also a reduction of biofilm of approximately 90% and destroyed 99% of the bacteria that were present in the mature biofilm. The action of endolysin was well highlighted, thanks to the use of a confocal microscope [79].

## 6. Phage Therapy in Swine Species

The first studies on the possibility of using phage therapy in pigs were conducted in the 1980s by Smith and Hugging [80]. After experimentally inducing diarrhea from *E. coli* O20: K101 987P in 7, the researchers treated the animals 13–16 h after infection with a phage mixture consisting of two bacteriophages (P433/1 and P433/2) concentration of 10^10^ PFU or with a monophagic preparation consisting only of P433/1. Diarrhea disappeared between 18 and 22 h after the phage treatment, while in the control group, several serious clinical signs were observed, not only ascribable to the gastrointestinal system alone, but the authors also described dehydration, ataxia, mental confusion. Feeding through a gastric tube prevented the animals in the control group from dying [80].

The positive effects of the use of phage therapy were also highlighted for the treatment of the enterotoxigenic *E. coli* strain O149: H10: F4 [81]. Before the study began, the animals were treated with florfenicol to facilitate the colonization of *E. coli* strains subsequently used for infection. The animals were infected orally with 10^10^ CFU of *E. coli* and subsequently were treated with six phages administered individually or as a multiphage mixture (phages GJ1-GJ7) at a dosage of 109 PFU for each phage. The phages were used both for preventive purposes (administered 15 min after infection) and for therapeutic purposes (24 h after infection). The duration and severity of the symptoms were reduced thanks to the preventive administration of the proposed phage therapy. Even the administration for therapeutic purposes has favored a remission of the symptoms in a shorter time, without any damage to the commensal *E. coli* strains normally present in the pig microbiota. Several studies also showed that the administration of phage therapy is safe, but it also has contributed to increased weight gain in the tested [82,83]. A research team assessed the effectiveness of using a phage cocktail consisting of 16 bacteriophages in the control of *Salmonella typhimurium* (y4232). The infection and the administration of the cocktail took place simultaneously. Through an oral gavage, the bacteriophages were contained in alginate beads in pigs 3–4 weeks old. The ileum, the blind, and the tonsils of the animals treated with the phage preparation had fewer bacteria from 2 to 3 log10 CFU/g comparing with the control group. In the same study, pigs that reached the slaughter weight were infected with 5 × 10^9^ CFU of *S. typhimurium* administered orally and treated with 10^10^ PFU of the microencapsulated phage mixture after two days from the infection (the administration was performed orally three times, observing an interval of 2 h between one treatment and the next). Again, the authors observed a reduction in the number of colony-forming units in the treated animals (1.4 log10 CFU/mL) compared to the control group [84].

An experimental study to evaluate the effectiveness of a phage cocktail was conducted on 21 pigs, divided into 3 groups (7 animals per group). A group was administered the microencapsulated cocktail together with food for five days, and subsequently, the animals were infected orally with *S. typhimurium*. The second group was treated with 60 mL of phage mixture orally, preceded by infection with *S. typhimurium*. The cocktail was administered 3 times in 2 h intervals. The last group, the control group, was only infected with *S. typhimurium* on the fifth day after the start of the experiment and did not receive any other therapeutic treatment. The phage therapy administered showed that the fecal elimination of the bacterium after 2 and 4 h was lower when the treatment was administered as a food additive compared to the control, while the group treated orally showed results superimposable on the control group. Furthermore, the bacterium count in samples of ileal and cecal content was also less than 1 log10 CFU/g in animals treated with the microencapsulated preparation administered with the food, compared to the control group [85]. The therapeutic efficacy of a multiphage preparation capable of lysing 34 reference strains and 99 isolated strains (out of a total of 107 tested strains) of *S. typhimurium* was tested in vivo on 4-week-old pigs. Pigs of 4 weeks of age were treated with the phage mixture for 15 days. On the seventh day of treatment, the pigs were infected with *S. typhimurium* (10^8^ CFU/mL in 10 mL). The results of the study were encouraging in the stool samples taken in the 7 days following the infection; the presence of *Salmonella* was not found, while in the control group, the colonies were 1.0 log10 CFU/mL [86].

*Yersinia enterocolitica* recognizes pigs as the main reservoirs of infection, and food from pigs is the most frequent source of infection for humans. The most frequent serotypes circulating in Europe are O: 3, O: 9, O: 5, and O: 8. In 2016, two double-stranded DNA bacteriophages were isolated: vB_YenM_TG1 (TG1) was isolated from pig manure in Canada and vB_YenM_ϕR1-RT (ϕR1-RT) from wastewater in Finland. These bacteriophages have a restricted host spectrum against O: 3, O: 5., and O:9 strains in conditions below 25 °C. An in vitro study assessed the possibility of using 4 bacteriophages (fHe-Yen3-01, fHe-Yen9-01, fHe-Yen9-02, and fHe-Yen9-03), which recognize *Y. enterocolitica* as the host bacterial cell. The bacteriophage fHe-Yen9-01 had a narrower host spectrum than the other 3 (active against 61.3% of the strains). To evaluate the suitability of the fHe-Yen9-01 bacteriophage against serotype O:9 Ruokola/71 strain, in a food model, the same group of research, after contaminating with *Y. enterocolitica* food samples of raw pork at 4 °C for 72 h, ready-to-eat meat products at 26 °C for 12 h and milk at 4 °C for 72 h, they treated the food samples with bacteriophage. The authors observed a 1 to 3 log decrease from the initial levels of 2–4 × 10^3^ CFU/g or mL. The authors also showed that in kitchen utensils, such as knives and cutting boards of various materials (wood and plastic), treated with the bacteriophage mixture, the bacterial growth decreased to 2 logs [87].

Respiratory diseases in swine are a serious problem in swine breeding. They are caused by various types of etiological agents, environmental problems and incorrect sanitary management. *Bordetella bronchiseptica* is considered a primary etiological agent responsible for swine respiratory disease (SRD), while *Pasteurella multocida* is among the secondary agents. Etiological agents often interact with each other and influence the duration and prognosis of the disease. *B. bronchiseptica* causes atrophic rhinitis and bronchopneumonia in pigs and predisposes animals to subsequent colonization of other viruses and bacteria responsible for secondary infections. Primary *B. bronchiseptica* infection is often responsible for secondary infection with toxigenic strains of *P. multocida*, which causes progressive atrophic rhinitis. Some authors have evaluated the possibility of using bacteriophages to control respiratory diseases in the pig by infecting PK75 pig nasal turbinate cells with *B. bronchiseptica*. The cells were infected with different concentrations (1 × 10^6^, 1 × 10^7^, and 1 × 10^8^ CFU/mL) and infection times (4 to 24 h) of *Bordetella* to evaluate the production of cytokines and mucin. The bacteriophage used was the lithic phage Bor-BRP-1 isolated from wastewater and feces from pig farms at a concentration of (1 × 10^7^ CFU/mL). The infection of PT-K75 cells with *B. bronchiseptica* resulted in an increased level of cytokines and chemokines, which are responsible for regulating the inflammatory response to the airways, as well as the expression of the Muc1 gene, which encode for the glycoprotein mucin, which protects the mucosa of several organs from pathogens invasion, was increased. In cells treated with bacteriophage, it was possible to observe that the production of cytokines and the expression of the Muc1 gene were, on the contrary, lower than in the control group cells. The bacteriophage against *B. bronchiseptica* is capable of inhibiting the production of cytokines and mucin, the overproduction, which is responsible for exaggerated inflammatory responses. This characteristic of the bacteriophage to regulate the inflammatory response has positive implications for the treatment of respiratory diseases in pigs [88,89].

Few studies were published on the possibility of using phage therapy in pigs and animal species in general in the fight against infections established by *P. multocida*. A recently published study has demonstrated the possibility of using phage preparations as an alternative to antibiotics. The study was carried out in vitro on pig nasal turbinate cells. The bacteriophage against *P. multocida* Pas-MUP-1 was applied before the infection of the cells with *P. multocida* (24 h before the infection). The cells were subsequently infected with 1 × 10^7^ CFU/mL of *P. multocida*. The inflammatory response was subsequently evaluated. As in the case of *B. Bronchiseptica* infection, PT K75 cells infected with *P. multocida* showed excessive production of IL-1β, IL-6, and Muc1. In the case of preventive treatment with the Pas-MUP-1 bacteriophage, the situation changed. In fact, a reduction in the production of inflammatory molecules was observed, specifically altering gene expression [90].

The possibility of using not only bacteriophages but also derived molecules, such as phage endolysins, was evaluated to combat infections caused by *E. rhusiopathiae*. Some authors investigated the antimicrobial potential of the LysP11 molecule, which is coded by *Propionibacterium* bacteriophage P1.1, which has no homology with the other endolysins produced by bacteriophages. Researchers produced an active LysP11 endolysin in N. benthamiana (a plant). This molecule has been observed to bind *E. rhusiopathiae* specifically, thus showing antibacterial activity against this pathogen [91].

## 7. Phage Therapy in Companion Animals

The number of pets in households has increased in the past 30 years and is steadily increasing, particularly in industrialized countries. The role of the dog and cat in our society has changed. Today greater attention is paid to the wellbeing and health of companion animals. Unfortunately, human–animal coexistence can also have negative effects. In addition, transmitting zoonoses, pets can also act as reservoirs for spreading antimicrobial resistance. Pets can be a vehicle for strains resistant to common antibiotic therapies, such as methicillin-resistant *Staphylococcus pseudintermedius* strains (also known as MRSA) or *Enterococci* that show resistance against vancomycin (VRE) [92,93]. Antibiotic-resistant strains are also commonly isolated from our pets, as well as constituting damage to their health, as conventional antibiotic therapies fail. They also pose a threat to public health. To ensure the health of animals, humans, and the environment, in the One Health perspective, responsible use of antibiotic molecules and the search for alternative drugs, such as bacteriophages, are necessary [94]. The literature on phage therapy in farm animals is quite extensive when compared to studies on the effectiveness of phage therapy in companion animals. Although scientific papers are rather sparse, studies published in dogs have provided encouraging results. The first description of the possibility of using phage therapy to control infections caused by *Pseudomonas aeruginosa* was published in 2006. In addition to reporting a human case with skin infections after burns, this article also describes the treatment of chronic otitis in a 5-year-old Saint Bernard dog. In addition to presenting chronic otitis, which affected both ears, caused by *P. aeruginosa*, the dog also had atopic dermatitis. Unfortunately, all conventional therapies with both systemic and locally applied antibiotics proved ineffective. The authors experimented with the use of bacteriophage-based therapy on both the human patient and the dog. The researchers observed that shortly after the application of the phage preparation, the virus started multiplying, which was more evident in the dog than the human patient. The article describes the evolution of clinical cases in little detail, and no details were reported regarding the phage preparation used. The treatment in the dog proved effective. The authors described that improvement was noticeable as early as one day after the phage treatment had been administered. Moreover, the absence of adverse reactions related to the administration of the phage preparation showed that it was also safe. In the following months, the presence of the pathogen was constantly monitored through the bacterial isolation of the exudate coming from the dog’s ears, and the animal achieved complete recovery after nine months of application of the phagic preparation without any antibiotic treatment [95]. A few years later, the first in vivo experimental study in dogs was published for the treatment of external otitis caused by *P. aeruginosa* through the use of phage therapy. The experimental study was conducted on 10 dogs that were naturally afflicted with external otitis caused by *P. aeruginosa*. To prepare the phage cocktail to be administered to animals from isolated phages, the researchers identified the phages, which were most suitable for killing the strains most commonly found during otitis in dogs. The phage combination chosen for the in vivo study was the one that had almost all of the *P. aeruginosa* strains were destroyed (90%). The phage cocktail consisting of 6 bacteriophages was administered locally, directly into the ear canal of the animals, at a dosage of 0.2 mL (1 × 10^5^ PFU). Before and after the phage treatment, several parameters were assessed, such as the clinical improvement of the animals and the pathogen count. During the treatment, on the other hand, the quantity of bacteriophage present in the dogs’ external ear canal was evaluated. The dogs were evaluated for the first time in the course of the experiment 48 h after the first administration of the phage cocktail. Moreover, after 2 days, the results were surprising: the local bacteriophages had multiplied by almost 100%, the increase in the bacteriophages had resulted in a reduction of about two-thirds of the bacterial load, and the clinical signs of external otitis had decreased by 30% [96]. The results of this in vivo study were so encouraging that shortly after its publication, a product based on bacteriophages for the treatment of *P. aeruginosa* otitis in the dog was put on the market, unfortunately now this product is no longer on the market. Recently, Furusawa et al. isolated 2 phages: ΦS12-1 and ΦR18. They tested their ability to lysate *P. aeruginosa* isolated from dogs. Their results showed the ability of the phages to lysate between (28/39) strains of *P. aeruginosa*, including the strains resistant to fluoroquinolones (4/6) [97].

*E. coli* is a microorganism that can cause a wide range of infections capable of affecting various organs and systems. In fact, pet and human urinary infections caused by this bacterium are not uncommon. A New Zealand research team assessed the in vitro efficacy of 40 bacteriophages against 53 uropathogenic *E. coli* strains. From the promising results obtained, more than 90% of the bacterial strains have been effectively lysed by phage cocktails; phage therapy could represent a valid alternative to conventional therapies in case of urinary infections in dogs and cats [98].

Recently, a research team has undertaken to isolate and characterize specific bacteriophages for MRSP strains, i.e., the methicillin-resistant *Staphylococcus pseudintermedius* strains, which increasingly prevent the therapeutic efficacy of antibiotic molecules in infections in dogs. The bacteriophages were isolated from the feces of dogs to evaluate the host spectrum and were tested on 66 *S. pseudintermedius* strains (17 strains resistant to methicillin, 43 sensitive to methicillin and 6 isolated directly from dogs). All phages showed lytic abilities against all resistant strains but were able to kill only a limited number of sensitive strains (16–28%) [99]. Table 1 summarizes the data discussed so far.

## 8. Regulatory Aspects

Scientific research in recent years has shown that bacteriophages can represent a viable alternative or be part of conventional antimicrobial therapy in combination with antibiotics. However, there are several aspects related to phage therapy that are unregulated. The lack of adequate legislation that regulates its use has as a consequence not only on the limitation of use but also indirectly affects the research, as the lack of legislation could discourage research in the field of phage therapy [100]. Bacteriophage-based treatments were administered following the Helsinki declaration formulated during the 8th World Medical Association general assembly (Helsinki, Finland) and dating back to 1964. The Helsinki declaration was approved over 50 years ago. It is obsolete and does not reflect current knowledge. Belgium, in 2001 (Directive 2001/83), with a regulation, approved the use of bacteriophages as an ingredient of an active pharmaceutical product in a magistral preparation. However, this directive has legal force only in Belgium, and these products are prepared in pharmaceutical laboratories under the supervision of the doctor and pharmacist [101]. Compared to conventional antibiotic molecules, enzyme proteins have different characteristics and should also require different testing methods. A specific regulation, unique in its kind, has been issued to regulate the use of Staphefekt, a lytic enzyme directed against *S. aureus*. It has been declared as a class 1 medical device by the European Union since 2013. It is a topical drug [102].

At present, although there is no specific legislation in the United States that regulates the use of bacteriophages. It is possible to treat patients with severe infections resistant to antibiotic therapies. It is necessary for the physician to make a specific request for phage therapy as an experimental therapy to the US Food and Drug Administration (FDA). The isolation, characterization, production of bacteriophages is very important for the FDA. It is based on this information that the FDA authorizes clinical studies to evaluate the efficacy and safety of the drug. Recently, the (FDA) authorized researchers from the University of San Diego and the biotech company AmpliPhi Biosciences Corporation to carry out a clinical study of a bacteriophage-based drug against *Staphylococcus aureus* to be administered intravenously [103]. The preclinical phase is of fundamental importance also for another important government agency, the US National Institute of Health (NIH), to identify and characterize phages that could be employed in drug development [104].

As for veterinary medicine, the situation is more complex compared to human medicine. When a drug is administered to animals, especially one that is intended for human consumption, a whole series of rules must be respected. There are some pharmacological categories that cannot be administered to farm animals. For other drugs, instead, before slaughtering the animal or collecting its milk, an appropriate withdrawal time of the drugs needs to be applied before licensing for human consumption. In addition, another aspect that veterinary medicine must take into consideration in addition to the protection of human and animal health is environmental protection. Unfortunately, there is no evidence showing the consequences of the incorrect disposal of phage preparations. The lack of this basic knowledge, probably dictated by the lesser interest in the application of phage therapy to veterinary medicine, translates into the fact that at the moment, there is still no European legislation concerning the use of bacteriophages or products derived from them in animals [4].

## 9. Conclusions

Although there are scientific documents that demonstrate bacteriophages’ discovery as early as the end of the 19th century, the possibility of using these viruses in antimicrobial therapy was assessed only in 1915. Due to the focus of interest by western medicine in antibiotics, eperiments with phage therapy continued in only a few centers in Eastern Europe. In recent years, the need to find new antimicrobial molecules to overcome the antibiotic resistance phenomenon has led to a rediscovery of bacteriophages also by western medicine. The greater knowledge of molecular biology techniques, the possibility of using experimental models, and the knowledge of bacteriophage–bacterial host interaction mechanisms have been very useful for a vigorous resumption of the studies in the field of phage therapy. The studies conducted so far showed that it is possible to use bacteriophages both as monophagic and multiphage preparations; it is also possible to use molecules derived from phages that have the advantage of being more stable, both alone and in association with antibiotics, although a very high dosage of antibiotics is needed to avoid the appearance of resistance. Many phage preparations can be administered with different administration routes, and strategies have been developed to prevent the preparations from being inactivated by gastric acidity or the action of digestive enzymes. However, the pharmacokinetics of bacteriophages is not entirely clear, although phage-derived enzymes would appear to have more stable pharmacokinetics. The diffusion of the phage to the blood after oral administration is very fast. In two to three hours, it can be found in the bloodstream and in 10 h in the organs. Antibodies production against phages is described. They can be an obstacle to the success of a phage therapy that appears especially after a second administration but can be easily solved by using repeated administrations or using different phages. Many other factors need to be taken into consideration for successful phage therapy, such as the proper isolation of the phages and their ratio with the target bacteria. The phage’s resilience to environmental factors, such as pH and temperature, are sure elements that affect phage replication and thus phage therapy. The texture where the target bacteria are localized plays an important role because some factors can shield the bacteria from the action of the phages. The resistance developed by bacteria through different mechanisms, such as loss of phage receptors or degradation of the nucleic acid of the phage, is a factor to be taken into consideration for the success of the therapy. Using a multiphage preparation or isolation of new phages can help to overcome this issue.

Regarding the possibility of using phage therapy in veterinary medicine, in the literature for some species, there is greater evidence of the use of phage therapy. In chickens, phage therapy is recommended especially for the control of the most frequent infections like salmonellosis, campylobacteriosis and colibacillosis, with extensive literature to support its use, the first experiments dating back to the beginning of the past century. In cattle, the possibility of the use of phage therapy for the prevention and control of mastitis, which is one of the most common and expensive pathologies for a farm, has been largely studied. All the data analyzed suggested that the use of phage therapy is mainly linked to fighting mastitis caused by *S. aureus*. Phage therapy was also extensively evaluated in swine farms. In particular, in this species were evaluated treatments with phage therapy for the control of most important zoonotic agent like *Escherichia coli* and *Salmonella enterica*, but also for the treatment of disease like swine respiratory disease (SRD) caused by *Bordetella Bronchiseptica e/o Pasteurella multocida* that affect the swine breeding from an economic point of view. Researchers only recently showed an interest in the possibility of using phage therapy in pets. Designing an in vivo experimentation on pets is rather complicated. While in most in vivo studies conducted on farm animals, infections were experimental, in the case of pets, they were not experimentally infected. Probably, due to these difficulties in conducting an in vivo experiment, the obvious ethical reasons can explain the few studies on the efficacy of phage therapy in vivo for pets. Finally, it is important to remember that bacteriophage–host interactions can provoke the appearance of phage resistance mechanisms. Therefore, if the use of phage preparations is authorized soon, they must be used responsibly to avoid making the same mistakes that led to the phenomenon of antibiotic resistance acquired.

## Figures and Tables

**Table 1:** Summary of the data analyzed in this manuscript (first column from the left: bacterial species analyzed in the study. Second column from the left: phage therapy utilized to reduce the bacterial species considered. Third column from the left: substrate used in the research to analyze the phages effect. Fourth column from left: reference number of the study).

Target Bacterial Species	Type of Phage Preparations Administrated	Animal Species or Cellular Substrate Used	References
*Bordetella bronchiseptica*	Monophage preparation (Bor-BRP-1)	Swine nasal turbinate cells	[88]
*Bordetella bronchiseptica*	Monophage preparation (Bor-BRP-1)	Swine nasal turbinate cells	[89]
*Campylobacter jejuni*	Monophage preparation(NCTC 12669 and NCTC 12671)	Chickens (one day old)	[58]
*Campylobacter jejuni*	Multiphage preparation(HPC5 and GHC8)	Chickens (25 days old)	[60]
*Campylobacter jejuni*	Multiphage preparation(F198, F287, F303, and F326)	Chickens (one day old)	[64]
*Campylobacter jejuni*	Multiphage preparation in different combinations (F198, F287, F303, and F326).	Chickens gut microbiota	[64]
*Campylobacter jejuni*	Multiphage preparation (CP1, CP14, F14, CP32, CP81, CP78, CP75, CP84, CP7; CP83, CP21)	Chickens (one day old)	[65]
*Campylobacter coli* and *Campylobacter jejuni*	Multiphage preparation (phiCcoIBB35, phiCcoIBB37, phiCcoIBB12)	Chickens (one day old)	[66]
*Clostridium perfringens*	Multiphage preparation (cocktail name INT-401)	Chickens (28 years old)	[68]
*Escherichia coli*	Monophage preparation (SPR02)	Chickens (3 days old)	[52]
*Escherichia coli*	Multiphage preparation(DAF6, SPR02)	Chickens (7 days old)	[53]
*Escherichia coli*	Multiphage preparation combined or not with enrofloxacin (DAF6 and SPR02)	Chickens (7 days old)	[54]
*Escherichia coli*	Monophage preparationSPR02	Chickens one day old	[56]
*Escherichia coli* (K1^+^ strain)	Monophage preparation(R)	Chickens (3 weeks old) and calves	[57]
*Escherichia coli*	Monophage preparation(CJ12)	Weaned pigs (3 weeks of age)	[83]
*Escherichia coli*	Multiphage preparation(phi F78E, phi F258E, and phi F61E)	Chickens (5 days of age)	[55]
*Escherichia coli*	Multiphage preparation(B44/1, B44/2, B44/3)	Calves, piglets and lambs (age not reported)	[80]
*Escherichia coli*	Mixture of 6 phages used alone or incombination (GJ1, GJ2, GJ3, GJ4, GJ5, GJ6, GJ7)	Weaned pigs (3 weeks of age)	[81]
*Salmonella*	Multiphage preparation (cocktail named BPT2) combined with antibiotics (apramycin) or not	Pigs (6 weeks of age)	[82]
*Pasteurella multocida*	Monophage preparation (Pas-MUP-1)	Swine nasal turbinate cells	[90]
*Pseudomonas aeruginosa*	Multiphage preparation(BC-BP-01, BC-BP-02, BC-BP-03, BC-BP-03, BC-BP-04, BC-BP-05, BC-BP-06)	Dogs (age not reported)	[96]
*Salmonella enterica* serovar *Enteritidis* (nalidixic acid-resistant strain)	Monophage and multiphage preparation (P1:1, CON, MOT2, IP, UDF1, YP, EP2, M4, MUT3, P22 hc2, P22 cPII, P22 cl-7, Felix O)	Chickens (14 days old)	[46]
*Salmonella enterica* serovar Enteritidis	Monophage preparation (PSE)	Quails (36 days)	[50]
*Salmonella enterica* serovar Enteritidis	Multiphage preparation(CNPSA1, CNPSA3 and CNPSA4)	Chickens (one day old)	[47]
*Salmonella enterica* serovar Enteritidis	Multiphage preparation(Φ151, Φ25, Φ10)	Chickens (34 days old)	[48]
*Salmonella enterica* serovar Enteritidis	Multiphage preparation(BP1, BP2, and BP3)	Chickens (10 days old)	[49]
*Salmonella enterica* serovar Typhimurium	Multiphage preparation(PEW 1, 2, 3, 4, 5, 6, 7, 8, 9, 10, 11, 12, 13 and 14)	Weaned pigs (3 weeks old) In market-weight pigs (about 110 kg)	[84]
*Salmonella enterica* serovar Typhimurium	Multiphage preparation(SEP-1, SGP-1, STP-1, SS3eP-1, STP-2, SChP-1, SAP-1, SAP-2)	Weaned pigs (3 week of ages)	[86]
*Staphylococcus aureus*	Monophage preparation (K)	Lactating dairy cattle (age not reported)	[72]
*Staphylococcus aureus*	Monophage or multiphage preparation (ΦH5, ΦG7, and ΦA72)	Lysogenized cells, milk	[73]
*Staphylococcus aureus*	Monophage preparation (SPW)	Bovine mastitis	[76]
*Staphylococcus aureus*	Multiphage preparation (STA1.ST29, EB1.ST11, and EB1.ST27)	Bovine mastitis	[77]
*Streptococcus dysgalactiae*, *agalactiae* and *uberis*	Phages endolysins λSA2 and B30	Bacteria in cow milk; mouse model	[78]
*Streptococcus agalactiae*	Bacteriophage lysin CHAP K	Milk	[79]
*Yersinia enterocolitica*	Multiphage preparation (fHe-Yen9-01, fHe-Yen9-02, and fHe-Yen9-03)	Food and kitchenware	[87]

## Data Availability

Data available in a publicly accessible repository. The data presented in this study are openly available in the Pub Med database.
